# Exploiting common senses: sensory ecology meets wildlife conservation and management

**DOI:** 10.1093/conphys/coab002

**Published:** 2021-03-29

**Authors:** Laura K Elmer, Christine L Madliger, Daniel T Blumstein, Chris K Elvidge, Esteban Fernández-Juricic, Andrij Z Horodysky, Nicholas S Johnson, Liam P McGuire, Ronald R Swaisgood, Steven J Cooke

**Affiliations:** 1 Fish Ecology and Conservation Physiology Laboratory, Department of Biology and Institute of Environmental and Interdisciplinary Science, Carleton University, Ottawa, ON K1S 5B6, Canada; 2 Department of Ecology and Evolutionary Biology, Institute of the Environment and Sustainability, University of California, Los Angeles, Los Angeles, CA 90095-1606, USA; 3 Department of Biological Sciences, Purdue University, West Lafayette, IN 47907, USA; 4 Department of Marine and Environmental Science, Hampton University, Hampton, VA 23668, USA; 5 USGS, Great Lakes Science Center, Hammond Bay Biological Station, Millersburg, MI 49759, USA; 6 Department of Biology, University of Waterloo, Waterloo, ON N2L 3G1, Canada; 7 Institute for Conservation Research, San Diego Zoo Global, San Diego, CA 92027-7000, USA

**Keywords:** Conservation, multidisciplinary, sensory ecology, sensory modality

## Abstract

Multidisciplinary approaches to conservation and wildlife management are often effective in addressing complex, multi-factor problems. Emerging fields such as conservation physiology and conservation behaviour can provide innovative solutions and management strategies for target species and systems. Sensory ecology combines the study of ‘how animals acquire’ and process sensory stimuli from their environments, and the ecological and evolutionary significance of ‘how animals respond’ to this information. We review the benefits that sensory ecology can bring to wildlife conservation and management by discussing case studies across major taxa and sensory modalities. Conservation practices informed by a sensory ecology approach include the amelioration of sensory traps, control of invasive species, reduction of human–wildlife conflicts and relocation and establishment of new populations of endangered species. We illustrate that sensory ecology can facilitate the understanding of mechanistic ecological and physiological explanations underlying particular conservation issues and also can help develop innovative solutions to ameliorate conservation problems.

## Introduction

Animals possess a variety of sensory systems that perceive salient features of the environment and facilitate critical, fitness-enhancing decisions (Dusenbery, 1992). Sensory systems thus evolved to allow animals to detect a variety of potentially important signals and cues such as light, sound, chemical, mechanical (Bradbury and Vehrencamp, 2011), magnetic (Wiltschko and Wiltschko, 2005) and electric (Bullock, 1973; Himstedt *et al*., 1982; Scheich*et al*., 1986) stimuli. Presence and acuity of sensory systems vary across species (Smith, 2008), allowing them to inhabit different environments or different functional niches within the same environment (Horodysky *et al*., 2010; Safi and Siemers, 2010). The field of sensory ecology studies how animals acquire, process and use sensory stimuli from their environment (Dusenbery, 1992). In the past decade, sensory ecology has seen an increase in research activity and associated literature, which has enhanced mechanistic understanding of animal behaviour (Stevens, 2013; Ruxton *et al*., 2018).

Insights from sensory ecology have important implications for conservation and management of wildlife with connections to the emerging disciplines of conservation behaviour (Buchholz, 2007; Blumstein and Fernández-Juricic, 2010) and conservation physiology (Cooke *et al*., 2013) and their intersection (Cooke *et al*., 2014; Horodysky *et al*., 2016). For example, by quantifying the range of stimuli that animals perceive, we can predict potential responses to environmental change, including urbanization and other human development, enabling better management decisions and informing future infrastructure designs to minimize harm to wildlife (Lim *et al*., 2008, Blumstein and Fernández-Juricic, 2010). In particular, as the human footprint expands across the Earth, sound and light pollution have increased and these can negatively affect animals by masking their natural sensory cues and signals or distracting and confusing them, potentially imposing negative fitness consequences (Halfwerk and Slabbekoorn, 2015; Dominoni *et al*., 2020). Increasing research focus on sensory mechanisms in focal species can provide vital information on how anthropogenic light or sound pollution can impact the decision-making processes of wild animals. Understanding the perceptual worlds of different species also helps prevent and ameliorate ecological and sensory traps and reduce human–wildlife conflict (Madliger, 2012) and has been beneficial in some instances at increasing the success rates of species translocations and re-introductions (Swaisgood, 2010). We can also exploit sensory perceptions to better manage target species as demonstrated by control of destructive, invasive species (Cruz *et al*., 2009; Johnson *et al*., 2009), predator and pest control (Maguire *et al.* 2009., Witzgall *et al*., 2010) and deterring animals from dangerous sites (Elvidge *et al*., 2018).

Although ecological research on visual and auditory senses has been conducted for nearly a century (Bayliss *et al*., 1936; Clarke, 1936; Hailman, 1977; Lythgoe, 1979; Neuweiler, 1989), only in the past two decades has the literature on sensory ecology expanded to include more taxa and sensory modalities with several important syntheses on the topic (see Endler and Basolo, 1998; Dangles *et al*., 2009; Stevens, 2013; Cronin *et al*., 2014; Martin, 2017). Similarly, an increasing amount of research is being conducted in applied sensory ecology, and we are seeing more case studies successfully applying this knowledge to aid in animal conservation or management. Several recent general reviews discuss the potential implications of sensory ecology for conservation biology (Madliger, 2012; Fernández-Juricic, 2016; Madliger *et al*., 2016; Dominoni *et al*., 2020), although most syntheses to date are species-, taxa- or sensory modality-specific (for example, Laiolo, 2010; Campbell-Palmer and Rosell, 2011; Martin, 2012; Jordan *et al*., 2013; Sorensen and Johnson, 2016). As the volume of primary literature and case studies of successful integration of sensory ecology and conservation science continue to grow, there is need for a more comprehensive review to understand the current state of knowledge and to inform future research and application.

**Figure 1:**
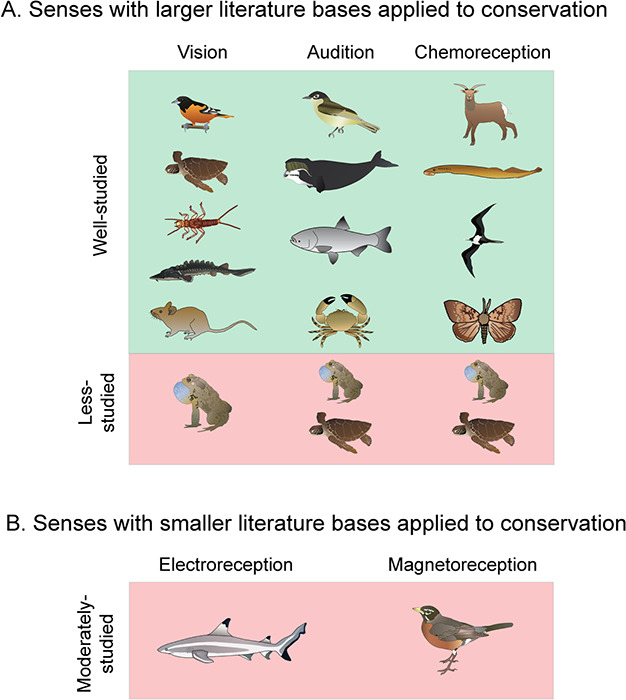
This figure provides a visual representation of taxa with known case studies demonstrating the application of sensory ecology to benefit wildlife conservation and management. Animals are categorized into six taxa: birds, mammals, invertebrates, fish, reptiles or amphibians. Each of these taxa is visually represented in the figure by a species from that taxon for which there has been a notable research demonstrating strong benefits of the integration of sensory ecology with conservation. Part (A) represents the three common senses, vision, audition and chemoreception, for which there is a larger amount of literature linking sensory ecology and conservation. Each of the six taxa specified by this paper are here categorized as either ‘well studied’ or ‘less studied’ depending on the presence or absence, respectively, of known literature linking sensory ecology to the conservation of species (or multiple species) of that taxon. Part (B) represents two sensory modalities, electroreception and magnetoreception, which have considerably smaller literature bases linking sensory ecology to conservation. However, we do note a single case study for each of these sensory modalities for which sensory ecology has benefited conservation of a species. Select Images by S. Bell, J. Hawkey, L. Fishman, K. Kraeer, and T. Saxby, courtesy of the Integration and Application Network, University of Maryland Center for Environmental Science (ian.umces.edu/symbols/).

Here, we present a comprehensive overview of the benefits to wildlife conservation and management that emerge from an understanding of sensory ecology and use the term ‘wildlife’ broadly to include all animal taxa. We review three major sensory modalities (vision, audition and chemoreception) as well as less understood modalities (electroreception, magnetoreception) and present case studies highlighting sensory ecology approaches relevant to conservation and management. In particular, we discuss case studies where sensory ecology has been demonstrated to successfully benefit a conservation problem (see Table 1), and also sensory ecology research that has furthered our understanding of certain conservation problems with potential to aid in the development of an innovative solution (see Table 2). Our review includes both vertebrate (mammals, birds, fish, reptiles, amphibians) and invertebrate studies where supporting literature is available and identifies major gaps in knowledge and avenues for future research (Fig. 1). We also discuss the importance of considering multimodal stimuli and some of the challenges associated with using sensory ecology knowledge to inform conservation and management.

**Table 1:** Summary of successful applications of sensory ecology in conservation and wildlife management

Sensory modality	Taxa	Species	Conservation issue solved	Overview	Reference
Vision	Bird	Canada geese (*Branta canadensis Linnaeus*)	Reduce airstrikes	Development of artificial lights to minimize collisions with aircraft	Blackwell *et al*., 2012
		Fairy terns (*Sterna nereis davisae*)	Relocation	Visual decoys attracted endangered fairy terns to safe breeding areas	Jeffries and Brunton, 2001
		Griffon vultures (*Gyps fulvus*)	Relocation	Cliff paintings that mimicked droppings attracted vultures to nest on suitable cliffs	Sarrazin *et al*., 1996
	Fish	White sturgeon (*Acipenser transmomtanus*)	Reduce entrapment and entrainment	Behavioural guidance of age-0 white sturgeon using coloured and strobing lights	Ford *et al*., 2018
	Invertebrate	Mayflies (Ephemeroptera), stoneflies (Trichoptera), dolichopodid dipterans, and tabanid flies (Tabanidae)	Ameliorate sensory trap	Fragmenting the solar-active area of solar panels reduced attractiveness of these panels to aquatic insects	Horváth *et al*., 2010
	Reptile	Green sea turtle (*Chelonia mydas*)	Ameliorate sensory trap	New dimmer, amber lights on Florida beaches reduced misguidance of sea turtle hatchlings away from the ocean	Witherington *et al*., 2014
		Green sea turtle (*Chelonia mydas*)	Reduce bycatch	Bycatch of turtles in commercial bottom gillnet fisheries reduced through use of LED and chemical light stick deterrents.	Wang *et al*., 2013
Audition	Bird	Black-capped vireo (*Vireo atricapilla*)	Relocation	Playing recordings of conspecific song attracted birds to more suitable habitats safe from brood parasitic species.	Ward and Schlossberg,2004
		European starling (*Sturnus vulgaris*)	Reduce airstrikes	Use of sound frequencies to create a ‘sonic net’ to deter birds from airfields	Swaddle *et al*., 2016
	Mammal	Harbour porpoise (*Phocoena phocoena*)	Reduce bycatch	Acoustic alarms successfully reduced bycatch of porpoises in gillnet fisheries, without reduced catch of target species	Larsen and Eigaard, 2014
	Fish	Asian carps (*Hypophthalmichthys nobilis, H molitrix Valenciennes*)	Invasive species control	Sound barriers effective at controlling spread of invasive Asian carps in the Great Lakes	Ruebush *et al*., 2012
		Multiple	Relocation	Playback of healthy reef sounds increased fish abundance and species richness in degraded coral reef habitat	Gordon *et al*., 2019
Chemoreception	Bird	Hooded plover (*Thinornis rubricollis*)	Reduce predation on threatened species	Conditioned taste aversion successful at reducing predation on threatened hooded plover eggs	Maguire *et al*., 2009
	Mammal	Common goat (*Capra hircus*)	Invasive species control	Female goats captured, sterilized and put in a chemically induced estrus to release pheromones to attract males for ineffective mating. Eradicated invasive goats from certain Galapagos Islands	Cruz *et al*., 2009
	Fish	Sea lamprey (*Petromyzon marinus*)	Invasive species control	Pheromone-based trapping highly effective, species-specific, method of capturing invasive sea lamprey in the Great Lakes	Johnson *et al*., 2009
	Invertebrate	Gypsy moth (*Lymantria dispar* L.)	Invasive species control	Invasive gypsy moths in the USA have devasting effects of forests that can be effectively controlled with pheromone traps	Tobin and Blackburn, 2007
Electroception	Fish	Hammerhead sharks (*Sphyrna lewini*)	Reduce bycatch	Commercial trawl fishing hooks made from electrorepulsive metals successful at reducing bycatch of sharks	Hutchinson *et al*., 2012

This table summarizes case studies where sensory ecology has been demonstrated to benefit conservation or wildlife management. We summarize relevant literature from all sensory modalities (vision, olfaction, chemoreception, electroreception and magnetoreception), across six major taxa (birds, mammals, fish, invertebrates, reptiles and amphibians) where valid case studies exist. We note that no relevant literature was found for amphibian species.

**Table 2:** Areas of sensory ecology research that have furthered our understanding of sensory issues, with potential to help solve conservation issues

Sensory modality	Taxa	Species	Overview	Reference	Conservation potential
Vision	Bird	Brown-headed cowbird (*Molothrus ater*)	Certain wavelengths are more likely to enhance detection and avoidance by individuals	Doppler *et al*., 2015; Goller *et al*., 2018	Using these lights on aircraft to reduce collisions.
		Various	Birds have more laterally projected vision, and this may contribute to increased collisions with human infrastructure	Martin, 2011	Warning signs placed on the ground before human infrastructure may be more beneficial to helping birds avoid collisions
	Mammal	Laboratory Wistar rats	Artificial light at night negatively affects sleep in rats	Stenvers *et al*., 2016	Increase our understanding of the negative impacts artificial light at night can have on various animals
		Tammar wallabies (*Macropus eugenii*)	Artificial light at night can affect reproductive timing of tammar wallabies	Robert *et al*., 2015	Increase our understanding of the effects of artificial light at night. Reduce artificial light at night during reproductive seasons of tammar wallabies
	Invertebrate	Various	Artificial light at night causes temporal disorientation, phototaxis and visual desensitisation	Owens and Lewis, 2018; Wakefield *et al*., 2016	Increase our understanding on this sensory trap. Possible solutions such as reducing the intensity or duration of artificial light at night, or using frequencies less attractive to insects
	Reptile	Green sea turtle (*Chelonia mydas*)	Sea turtles are more likely to consume plastic waste that resembles their common prey	Schuyler *et al*., 2014	Increase our understanding of this sensory trap. Reduce plastic waste in ocean
Audition	Bird	Western bluebird (*Siatia Mexicana*)	Noisy gas compressor stations increase physiological stress and reduce hatching success compared with quieter sites	Kleist *et al*., 2018	Use of newer or quieter compressor stations or using improved sound insulation at compressor sites
		Ovenbird (*Seiurus aurocapilla*)	Noisy compressor sites reduce mating success by dampening conspecific calls	Habib *et al*., 2007	Use of newer or quieter compressor stations or using improved sound insulation at compressor sites
		Various	Roadway traffic noise alone compromises avian density and overall body condition of birds in the area	Ware *et al*., 2015	Increase our understanding of the impacts of noise pollution
	Mammal	Pinnipeds	Acoustic deterrents effective at deterring pinnipeds from fish farms and fisheries	Götz and Janik, 2013	This knowledge could be used in a conservation context to prevent bycatch, or vessel collisions, of pinnipeds
		Brazilian free-tailed bat (*Tadarida brasiliensis*)	Activity of bats was lower at noisy compressor sites compared with quieter stations	Bunkley *et al*., 2015	Use of newer or quieter compressor stations or using improved sound insulation at compressor sites
		Greater mouse-eared bats (*Myotis myotis*)	Smooth vertical building surfaces can act as acoustic mirrors to echolocating bats, resulting in collisions	Greif *et al*., 2017	Redesign of certain buildings making them more ‘visible’ to echolocating bats
		Killer whales (*Orcinus orca*)	Noise from shipping traffic is a chronic stressor for killer whales and can disrupt foraging behaviour, pod communication, and echolocating clicks to detect prey	Williams *et al*., 2019b	Re-routing ships to avoid major whale habitat, use of quieter ships, reducing speed at which ships travel near important whale habitat
	Fish	Ambon damselfish (*Pomacentrus amboinensis*)	Ambon damselfish physiologically stressed by motorboat noise and show reduced ability to evade predators	Simpson *et al*., 2016	Banning motorboats from certain areas of important damselfish habitat
	Invertebrate	Zooplankton	Seismic studies used to detect petroleum in the ocean can cause significant mortality of zooplankton	McCauley *et al*., 2017	Further our understanding. Reduce the number of seismic studies conducted in the ocean
		Field crickets (*Gryllus bimaculatus*)	Traffic noise distracted female field crickets meaning they did not orient to male auditory calls	Schmidt *et al*., 2014	Further our understanding
		Hermit crab (*Coenobita clypeatus*)	Sound of motorboat noise distracts crabs and modifies their risk assessment allowing humans to get closer before retracting in their shells	Chan *et al*., 2010	Banning motorboats from certain areas of important crab habitat
		Squid (*Doryteuthis pealeii*)	Pile driving sounds elicited body pattern changes, inking, jetting and startle responses	Jones *et al*., 2020	Further our understanding. Future consideration in placement of wind farms to avoid important squid habitat
Chemoreception	Bird	Seabirds (order: Procellariiformes)	Marine plastics release DMS, which is also a common odorant released by the prey of many seabirds. Resulting plastic ingestion can lead to mortality	Savoca *et al*., 2016	Potential to synthesize plastics without chemical compounds attractive to seabirds or other marine life
	Mammal	Harvest mice (*Micromys minutus*)	Captive breeding improved in mice by familiarizing females with male scents	Roberts and Gosling, 2004	Proof of concept. This technique could be used to improve breeding success of endangered species, or species in captivity
		Pygmy loris (*Nycticebus pygmaeus*)	Urine scent has been shown to influence mate choice in threatened female pygmy loris	Fisher *et al*., 2003	This knowledge can be used to promote more beneficial mate pairing in this threatened species. Proof of concept—this technique could be used to promote more beneficial mate parings in other endangered species
	Fish	Sheephead swordtail fish (*Xiphophorus birchmanni*)	Water polluted by sewage effluent chemicals has been shown to reduce female preference for male olfactory cues	Fisher *et al*., 2006	Further our understanding of the negative impacts of water pollution on fish species, which could aid in stressing the need to reduce sewage effluent in natural aquatic habitats
	Invertebrate	Spanish moon moth (*Graellsia isabellae*); Valley elderberry longhorn beetle (*Desmocerus californicus dimorphus*); Rare click beetle (*Betarman bisbimaculatus*)	Pheromone trapping effective at attracting endangered species	Millar *et al*., 2010; Ray *et al*., 2014; Konig *et al*., 2016	Potential to use pheromone trapping as an effective way at monitoring populations of endangered invertebrates
Magnetoreception	Bird	European robin (*Erithacus rubecula*)	Electromagnetic noise from human activity disrupts magnetic compass orientation	Engels *et al*., 2014	Investigate use of potential electromagnetic frequencies that do not disrupt magnetic compass orientation
	Invertebrate	Edible crab (*Cancer pagurus*)	Electromagnetic field emissions attracted crabs to underwater cable sites and reduced foraging behaviour	Scott *et al*., 2018	Consideration of the location of underwater cable routes in areas of important crab habitat
Multisensory	Fish	Sea lamprey (*Petromyzoa marinus*)	‘Pull’ of attractive light frequencies and ‘push’ of odor repellents effective at controlling juvenile sea lamprey movement	Johnson *et al*., 2019	Potential for more effectively controlling movement of sea lamprey to either (a) protect individuals from entrapment/entrainment, or (b) control spread of invasive lamprey in certain areas

This table summarizes sensory ecology research that has furthered our understanding of certain conservation issues, with potential to help develop innovative solutions to these problems. Although the case studies summarized here have not been demonstrated to benefit conservation practices, we do discuss potential ways in which the knowledge could be applied for the benefit of conservation and wildlife management. We summarize relevant literature from all sensory modalities (vision, olfaction, chemoreception, electroreception and magnetoreception), across six major taxa (birds, mammals, fish, invertebrates, reptiles and amphibians) where appropriate research exists. We note that no relevant literature was found for amphibian species.

## Vision

Vision as a sensory modality can be defined not only as the ability to detect and respond to light stimuli, but also the ability to detect spatial structure and form an image (Stevens, 2013). Light is electromagnetic radiation exploited by many animals in the form of visual cues and signals (Stevens, 2013). Composed of photons travelling in waves, different types of electromagnetic radiation are grouped into functional categories by wavelength, ranging from very short and relatively high-energy (gamma and x-rays) to long and relatively low-energy (radio waves and microwaves) along the electromagnetic spectrum. Species, and even individuals, vary in the wavelength of light they are able to detect. Visible light, as defined by human detection ability (400–700 nm), is very roughly intermediate in the spectrum, yet photoreceptors in animal eyes are often sensitive enough to respond to wavelength differences between captured photons and perceive differences as colour. Beyond the light spectrum visible to humans, ultraviolet and infrared visual sensitivity is employed by numerous other species across different taxa. Photons also vary in properties beyond wavelength, notably the direction of their vibrating electric fields. All photons travel with a vibrating electric field that is perpendicular to the direction of motion or propagation, and the orientation of this electric field to the axis of propagation is referred to as the e-vector angle (Johnsen, 2012). Natural, unpolarized light consists of photons all with different e-vector angles, whereas polarized light consists of photons that all have mostly the same e-vector angle. While many species cannot perceive polarized light, others are capable of detecting it to inform spatial orientation, including some birds, fishes, reptiles, amphibians and both terrestrial and aquatic invertebrates (Cronin *et al*., 2003; Douglas *et al*., 2007).

In this section we review visual ecology research for five major taxa (birds, reptiles, fish, invertebrates and mammals) that has benefited conservation and wildlife management. Amphibian vision has been relatively understudied for the context of conservation (Fig. 1), and thus we do not cover this animal class here. Understanding what various species perceive has been important in understanding certain conservation issues and sensory traps, for example bird collisions, turtle hatchling misguidance and aquatic insects mistaking solar farms and roadways for water. Furthering our understanding of these visual traps has led to innovative solutions to these problems, as we will highlight through various case studies. Exploiting species vision has also proven to be beneficial to species relocation and translocation efforts, as well as guiding animals around, or alerting them to, particular hazards in their environment. Throughout this section we discuss case studies of successful application of visual ecology knowledge that has proven beneficial to a particular conservation problem (Table 1), as well as knowledge that has highlighted a particular conservation problem and has potential to lead to an effective solution (Table 2).

### Birds

Birds are visually oriented animals whose cone photoreceptor cells also have pseudoorganelles (oil droplets) that can enhance color discrimination (Martin, 2017). Color perception in birds is then a function of the spectral sensitivity of their visual pigments as well as the absorbance properties of their oil droplets (Martin and Osorio, 2008), and it has been suggested that avian color perception may vary considerably among species (Hart and Hunt, 2007). This variation poses a challenge for using visual beacons (e.g. LED lights) to prevent different bird species from colliding with human infrastructure (including buildings, wind turbines, aircraft, etc.). It has been estimated that millions of birds collide with buildings annually (Machtans *et al*., 2013) and thousands are reported to collide with aircraft (Dolbeer *et al*., 2015). However, artificial lighting can help prevent these collisions and some strategies to standardize the development of visual deterrent beacons have been implemented (Fernández-Juricic, 2016) by (a) characterizing key properties of the visual system in species with high frequencies of collisions, (b) including information of these visual properties on avian visual models to predict which wavelengths may be most stimulating to retinal photoreceptors and (c) conducting behavioral studies to assess which of these most-stimulating light colours can lead to changes in obstacle detection and avoidance behaviors. More specifically, these strategies have been applied to the development of aircraft running lights to minimize bird–aircraft collisions (Blackwell *et al*., 2012; Blackwell and Fernández-Juricic, 2013).

Sensory physiology has allowed for the characterization of the brown-headed cowbird (*Molothrus ater*) visual system (Blackwell *et al*., 2009; Fernández-Juricic *et al*., 2013), which subsequently allowed researchers to develop potential aircraft lighting to increase detection and avoidance by this species. Researchers have developed species-specific visual models (Goller *et al*., 2018), which yielded four wavelengths with high chances of stimulating the retinal cells of brown-headed cowbirds (380 nm, 470 nm, 525 nm and 630 nm, roughly corresponding to ultraviolet, blue, green and orange, respectively). In turn, behavioral studies have shown that LED lights with a 470 nm (blue) peak led to quicker visual detection (Doppler *et al*., 2015) and avoidance behavior (Goller *et al*., 2018). Collisions between birds and aircraft are not only often fatal to the bird but also can cause damage to aircraft thus representing a threat to public safety (Dolbeer, 2006), and aircraft running lights optimized for bird detection and avoidance offer great potential for reducing such collisions. Similarly, furthering our knowledge on avian vision has also helped understand bird–building collisions. Birds often focus on reflections of vegetation in the glass of buildings and are also known to have more lateral vision focused towards the ground (Martin, 2011), which can lead to collisions. As a result of this knowledge, certain cities such as New York City (NYCAS, 2007), Toronto (CTGDS, 2007) and San Francisco (SFPD, 2011) have released guidelines for bird-friendly building designs to minimize collision.

Avian vision can also be exploited to aid in the successful relocation of endangered species. Visual conspecific decoys have been used to successfully attract endangered fairy terns (*Sterna nereis davisae*) to safe breeding areas (Jeffries and Brunton, 2001), and painting rocks to mimic faecal droppings was successful at attracting griffon vultures (*Gyps fulvus*) to nest on cliffs that had not been chosen as nesting sites by this species for ~60 years (Sarrazin *et al*., 1996). Such manipulations may be effective in certain situations for social animals that use visual cues to detect conspecifics, and the efficacy of such manipulations has been formally reviewed (Putman and Blumstein, 2019).

### Mammals

Although mammals typically view the world through the ‘visible’ range of light, some species are also cabable of detecting ultraviolet wavelengths. For example the Arctic reindeer (*Rangifer tarandus*) that exploits ultraviolet vision to search for lichens and other food sources in dark winter periods (Hogg *et al*., 2011). Other mammalian species have also evolved eyes better adapted to visualizing their environments, such as marine and aquatic mammals (Mass and Supin, 2007) and nocturnal mammals, which typically have evolved larger eyes to capture more light in dark environments (Hall *et al*., 2012). Nocturnal mammals can be negatively affected by artificial light at night. In mammals, like most other taxa, the daily light–dark cycle is responsible for synchronizing the internal circadian clock, which is responsible for many metabolic, and ultimately behavioural, functions. Although there has been a great amount of research on mammalian vision, to our knowledge there has been very little application of mammalian visual ecology to benefit conservation effects to date. Therefore in this section we focus on research highlighting the effects of artificial light at night on noctural animals, and discuss the potential of this knowledge for conservation purposes.

Artificial light at night has been shown to increase body mass in mice (laboratory Swiss–Webster mice; Fonken *et al*., 2010), affect sleep in rat species (laboratory Wistar rats; Stenvers *et al*., 2016) and affect reproductive timing in wild tammar wallabies (*Macropus eugenii*: Robert *et al*., 2015). These behavioural changes can all negatively affect fitness and may therefore have population-level effects. More research into the effect of light at night on various species helps understand to what extent this light pollution is having across all species, which will further emphasize a need to better regulate light at night or help us develop innovative solutions to more environmentally friendly night lighting. Interestingly, there have been some beneficial effects of artificial lighting on certain mammalian species. Artificial light at night can cause phototaxis for a number of invertebrate species including moths (as will be discussed later in this section). Nocturnal feeding mammals can take advantage of this higher density of invertebrates around night lighting. One study found moth consumption of Cape serotine bats (*Neoromicia capensis*) under night lighting conditions to increase 6-fold compared with unlit conditions (Minnaar *et al*., 2015). In this instance artificial light at night is beneficial to the predator species but leaves the prey species more vulnerable to consumption.

### Fish

Most fish species have well-developed eyes, evolved to allow them to effectively see in subaqueous environments (Guthrie, 1986; Wagner, 2011). Even in deep sea bathypelagic zones, (beyond 1000 m in depth) where the only light present is that which emanates from bioluminescent animals, bathypelagic fish species have well-developed eyes (Landgren *et al*., 2014). Beyond the visible light range, certain fish species are capable of detecting ultraviolet (Flamarique and Hawryshyn, 1998, Smith *et al*., 2002) and infrared radiation (Meuthen *et al*., 2012). Research into fish vision has shown that certain light features can act as attractants or repellants for various species of fish, and this knowledge can be successfully exploited to help protect fish by repelling or guiding them away from harmful obstacles in waterbodies. Visual guidance in fish can therefore be an effective tool in conservation and wildlife management.

It has been known for some time that white or mercury vapour light (a high intensity discharge lamp) can be an effective repellant or attractant for various fish species (Haymes *et al*., 1984; Patrick *et al*., 1985), and strobe lighting mechanisms can be effective at guiding fish around human infrastructure such as dams to prevent entrapment or injury (Brown, 2000). However, the responses to various light wavelengths are species specific, which can cause problems when using light to guide or repel fish to or from certain areas. Fish species can have different capabilities in the detection and processing of visual stimuli (Horodysky *et al*., 2008; Horodysky *et al*., 2010; Morshedian and Fain, 2015). This is important to know when attempting to use visual cues as attractants or deterrents (Elvidge *et al*., 2019) or to evaluate the efficacy of bycatch reduction strategies without reducing target catch. In this context, sensory physiology has been used to determine peak sensitivities of various fish species based on the absorbance properties of the visual pigments in the retina (Sillman *et al*., 2007). Additionally, the prediction of peak sensitivities and subsequent behavioural assays have determined fish response to these light frequencies. For example, studies have documented peak sensitivity and positive phototaxis of white sturgeon (*Acipenser transmontanus*) to green, red and blue light (Sillman *et al*., 2007; Ford *et al*., 2018), both positive and negative phototaxis in response to different colours in lake sturgeon (*Acipenser fulvescens*: Sillman *et al*., 2007; Elvidge *et al*., 2019), and negative phototaxis in response to blue light in the American eel (*Anguilla rostrata*: Elvidge *et al*., 2018). Here, an understanding of sensory physiology leads to a better understanding of behavioural responses to different portions of the light spectrum. This could be highly beneficial in many circumstances, including attracting fish towards fishways enabling them to bypass dams or other obstacles, or in deterring fish from harmful infrastructure such as hydroelectric turbines or boat propellers, however, differences in species-specific responses to various wavelengths must be well considered.

### Invertebrates

There is a large amount of literature and research on insect vision, particularly for model species such as *Drosophila melanogaster* (Borst, 2009). Insects have anatomically and physiologically different eyes than vertebrates (Borst, 2009); however, most insects do have well-developed eyes and rely heavily on their vision. Some insect species are capable of detecting ultraviolet radiation, such as butterflies (*Bicyclus anynana*) for selecting mates (e.g. Lyytinen *et al*., 2004), and others can detect infrared radiation, such as black fire beetles (*Melanophila acuminata*), which use infrared to detect forest fires from distances indicating suitable low-risk places for females to lay eggs (e.g. Schmitz and Bleckmann, 1998). Certain insects also have eyes adapted for noctural vision (Warrant, 2017) and a number of species (in particular aquatic species) are capable of detecting polarized light and this has important implications for their survival. However, human development has led to several visual traps, such as artificial light at night, solar farms and roadways, which negatively impact certain insect species.

Solar farms and roadways can reflect polarized light, and for certain aquatic insects, their vision perceives these reflected light sources as a water surface on which they can lay their eggs (Schwind, 1995; Horváth *et al*., 2010). This knowledge has led to better solar panel designs to reduce their attractiveness to aquatic insects. One study was successful at reducing the attractiveness of solar panels by 10- to 26-fold by fragmenting their solar-active area with white partitions (Horváth *et al*., 2010). Artificial light at night has also been shown to have negative effects on certain insects, including spatial and temporal disorientation, attraction through positive phototaxis and visual desensitization due to high illumination (Grubisic *et al*., 2018; Owens and Lewis, 2018). A greater understanding of these sensory issues could lead to innovative solutions such as reducing the intensity or duration of lights at night, or using light frequencies that are less attractive to insects (Wakefield *et al*., 2016).

### Reptiles

Vision is important for many reptile species, which can differ in their retinal physiology and morphology to adapt to diurnal, nocturnal or crepuscular activity (Katti *et al*., 2019). Reptiles can detect electromagnetic radiation from ultraviolet (Kawamura and Yokoyama, 1998) to infrared wavelengths (Gracheva *et al*., 2010). Research into the sensory ecology of reptile vision has been important in conservation, particularly of certain turtle species. There are a number of conservation issues for turtle species resulting from visual traps due to anthropogenic activity, such as plastic ingestion in marine environments, misguidance of turtle hatchlings and bycatch in commercial fisheries.

Turtle visual ecology research has offered insight and further understanding into a common sensory trap for marine wildlife: plastic ingestion. Sea turtles are known to be vulnerable to plastic waste because turtle vision may perceive certain plastics as prey (Fritts, 1982). Plastic bags or balloons can be confused with jellyfish, a common prey species for sea turtles, and research has suggested that turtles are more likely to consume waste that resembles their prey (Schuyler *et al*., 2014). When ingested, the plastic cannot be digested and may have consequences including physical blockage of the digestive system, often resulting in death (Wilcox *et al*., 2018). The amount of plastic waste in the ocean is expected to keep increasing (Jambeck *et al*., 2015) and it has been estimated that 52% of all sea turtles globally have ingested plastic waste of some kind (Schuyler *et al*., 2015). Plastic ingestion can therefore become a serious threat to the conservation of wild sea turtles and furthering our knowledge of this sensory trap can help highlight the dangers of plastic waste to wildlife and promote actions to reduce plastic waste in our oceans.

We also note two case studies demonstrating the success of turtle visual ecology in increasing conservation efforts. For green sea turtle hatchlings (*Chelonia mydas*), seaward migration following emergence occurs predominantly during the night and hatchlings rely on light from the horizon above the sea to guide them to water (Lohmann *et al*., 1997). Artificial light at night has been demonstrated to misguide sea turtle hatchlings as they emerge from nests on beaches and attempt to navigate towards the ocean (Thums *et al*., 2016). These artificial lights are now the brightest light source on Wobiri Beach (North West Cape, Western Australia) and mask the light from the sea horizon, thus causing hatchlings to orient away from the sea which greatly reduces survival. Because sea turtle hatchlings are particularly vulnerable to disorientation from artificial light at night, new management initiatives have been implemented on Florida beaches (Witherington *et al*., 2014). In these areas, typical streetlights have been replaced with dimmer amber lights that are directed downwards instead of outwards toward the nesting sites. This initiative appears to be successful as after 1 year hatchling mortality from disorientation decreased significantly and remained low in following years (Witherington *et al*., 2014). Visual ecology of green turtles has also been beneficial for protecting adults, as well as hatchlings. A large amount of research into sea turtle sensory biology has been focused on reducing bycatch in fishing lines and nets (Horodysky *et al*., 2016). One trial successfully reduced bycatch rates of green sea turtles in commercial bottom gillnets (targeting fish) by 40% and 60% through use of LED and chemical light stick visual deterrents, respectively (Wang *et al*., 2013). No significant effect on target species catch rate or catch value was found when using these visual deterrents, making this a viable option for use in commercial gillnet fisheries to help reduce bycatch of sea turtles.

## Audition

Auditon, more commonly known as hearing, can be defined as the detection of acoustic stimuli (vibrations transmited through a medium; Pollack *et al*., 2016). Acoustic stimuli is typically referred to as ‘sound’ when these vibrations occur in fluid mediums (air or water), and as ‘substrate vibrations’ in a solid medium (Windmill and Jackson, 2016). In this paper we will use these definitions of sound and substrate vibrations. Vibrational waves of acoustic stimuli can vary in frequency, wavelength and intensity (Stevens, 2013). Frequency is defined as the number of wave cycles of a particular sound that occur per second, and thus is directly related to wavelength and defines the pitch of a sound. Sound intensity is energy transported by a sound wave and can be perceived as volume by a receiver (Lefebvre, 1999). Most animals have sensory organs that are capable of detecting a range of acoustic stimuli. Vertebrate hearing is often associated with the ear, a sensory organ with many features that allow it to detect certain sounds and propagate this information to sensory neurons (Ruggero *et al*., 1992; Ren, 2002). However, for certain species of fish and amphibians sound detection is also associated with specialized neuromast cells also capable of detecting acousti stimuli (Fay and Popper, 1998). Invertebrates also have sensory receptors capable of detecting a broad range of sound frequencies (Pollack *et al*., 2016). Sound stimuli can be detected by animals for a variety of purposes from prey detection and predator avoidance, mating and breeding and social interactions and communication. Although relatively understudied when compared with sound, substrate vibrations in animal communication are more exploited than once thought (Hill, 2001). Substrate vibrations have been shown to be important for a wide range of purposes such as predator–prey interactions, foraging, mate choice and breeding and materal care (Hill, 2001; Hill, 2009), and in all taxa from bees (Kirchner, 1997) to elephants (O’Connell-Rodwell *et al*., 2001). Some animals are also capable of producing sound frequencies for the specific purpose of detecting the echoes of this sound. This is a form of active auditon, known as echolocation, commonly used by cetaceans and bats (Jones, 2005).

In this section we review case studies of how auditory ecology has benefited wildlife conservation and management across four major taxa (birds, mammals, fish and invertebrates). Reptile and amphibian auditory ecology has been relatively understudied in the context of conservation, and thus we do not discuss these taxa in this section (Fig. 1). Anthropogenic development and activity ultimately creates unnatural sounds and vibrations in the environment, which can have many negative effects of various animal species (Slabbekoorn *et al*., 2018). From a conservation perspective, it is important to identify and understand the seriousness of these effects on animals in order to act to mitigate these problems. Auditory stimuli have also been shown to be effective at both attracting animals to certain habitats (e.g. through conspecific cues and signals to aid in species relocation), and also at deterring animals from habitats (e.g. certain sound frequencies have been effective at deterring animals from dangerous environments), which can both be beneficial to conservation and wildlife management.

### Birds

Avian species often rely heavily on hearing for a number of behaviours including hunting/foraging, predator avoidance, territorial defense and conspecific attraction for reproductive purposes (Winkler, 2001). Avian hearing is typically restricted to lower frequencies (below 10 kHz) and they cannot detect ultrasonic frequencies (above 20 kHz; Köppl, 2015). However, some bird species, such as pigeons, chickens and guinea fowl, are capable of infrasonic hearing (frequencies below 20 Hz) for purposes such as long-range detection of auditory cues from landmarks or weather events (Hagstrum, 2000; Zeyl *et al*., 2020). Some bird species, such as oilbirds and swiftlets, are even capable of echolocation to detect food such as fruits and insects, respectively (Brinkløv *et al*., 2013).

Anthropogenic development and activity can result in loud, unnatural sounds (‘sound pollution’), and this can have a number of negative consequences for bird species in many contexts (Ortega, 2012). For example, noisy natural gas compressor stations in New Mexico, USA, caused significantly increased levels of the stress hormone corticosterone in a community of nesting western bluebirds (*Sialia mexicana*) and a significant reduction in hatching success in noisy sites compared with control, quiet sites (Kleist *et al*., 2018). Sound pollution from compressor sites has also been shown to reduce mating success by dampening conspecific calls of ovenbirds (*Seiurus aurocapilla*: Habib *et al*., 2007). In one study, the sound of roadway traffic alone was enough to compromise both avian density and condition. Ware *et al*. (2015) created a ‘phantom road’ by amplifying traffic noise in a rural habitat with no road. The results of this study showed that the sound of traffic alone caused 31% of individuals of various species to avoid the area, and those that remained despite the noise had reduced overall body condition (a size-adjusted metric of body mass that signifies energy stores birds need for migration). Further examples show sound pollution can alter bird song (Gentry *et al*., 2018) and alter bird song timing (Nordt and Klenke, 2013). For example, shifting European blackbird (*Turdus merula*) song to earlier hours of the morning to avoid rush hour traffic causing sleep deprivation for the bird (Nordt and Klenke, 2013).

Conservation practitioners can also exploit bird auditory ecology to influence habitat selection behaviour to guide birds to settle in more appropriate areas. In a study by Ward and Schlossberg (2004), the black-capped vireo (*Vireo atricapilla*), a territorial songbird, was attracted to suitable habitat sites that were uninhabited by the species by playing recordings of the bird’s song. The researchers were successful at attracting birds to the experimental sites where the bird song was played, compared with control sites where no black-capped vireos were attracted over the same time period. In this example, the researchers attracted birds to sites where the brood-parasitic brown-headed cowbird (*Molothrus ater*) was controlled, and thus higher nesting success was seen compared with nearby black-capped vireo populations in uncontrolled habitats (Ward and Schlossberg, 2004). Similar studies with other songbirds have also proven successful, with 12/14 species in which playback of bird song were tested successfully attracting birds to settle in the area (Ahlering *et al*., 2006). Here we see how avian auditory ecology can be exploited to attract bird species to more suitable, safe habitats. However, we can also exploit avian auditory ecology to deter bird species from dangerous, unfavourable habitats. Airfields are an area with increased numbers of airstrikes between birds and aircraft. One study demonstrated that spatially controlled sound frequencies emitted around an airfield (a ‘sonic net’) were able to successfully deter birds from the area (Swaddle *et al*., 2016). These sound frequencies were chosen to overlap with the frequency range of the European starling (*Sturnus vulgaris*), and results showed an 82% reduction in the number of starlings at airfields with sonic nets compared with control areas. Previously we discussed how avian vision can be exploited to make aircraft more visible to birds to reduce airstrikes, and perhaps using a combination of approaches, and targeting more than one sense (multisensory approaches), may be more effective at either repelling species from unsuitable dangerous habitats, or attracting species to more suitable habitats.

### Mammals

Mammalian species are capable of producing and detecting a wide range of sound frequencies, from high-frequency bat calls (Manley, 2012) to low frequency whale songs (Darling, 2015). Mammals use auditory cues and signals for mating, hunting, predator avoidance, foraging and social communications (Suthers, 1978). Several mammals, most notably species of bats and toothed cetaceans, rely on echolocation to hunt prey, as well as to navigate in low light conditions (Thomas *et al*., 2004). Sound pollution affects echolocation in pallid bats (*Myotis myotis*), potentially interfering with signal reception and processing. Natural gas compressor stations reduce pallid bat activity levels by as much as 40% at louder stations compared to quieter ones (Bunkley *et al*., 2015). Similarly, frog-eating fringe-lipped bats (*Trachops cirrhosus*) can shift to active echolocation from passively listening for frog vocalizations when anthropogenic noise is present (Gomes *et al*., 2016). Noise pollution can also interfere with acoustic communication if it masks acoustic signals, reducing the ability of animals to coordinate socially for mating, territoriality, and other behaviours. In the case of the endangered Stephens’ kangaroo rat (*Dipodomys stephensi*), traffic noise not only masked foot-drumming signals but also served as an acoustic model that kangaroo rats appeared to mimic (Shier *et al*., 2012). This behavior may have important biological and fitness consequences compromising conservation. For example, noise pollution might cause distraction from true conspecific signals (lost mating opportunities), attraction to dangerous roads (mortality risk) or increased stress (may lead to decreased body condition and all the other problems with stress).

Sound can also travel through water, and sound pollution from boats and aquatic infrastructure has many negative impacts on marine mammals (Popper and Hawkins, 2012). Noise from shipping traffic is a chronic habitat-level stressor for many species of whales, including killer whales (*Orcinus orca*: Williams *et al*., 2019b). For a population of southern resident killer whales, shipping noise disturbance is thought to be one of the three main stressors responsible for declining population numbers, along with lower numbers of prey (salmonid fishes), and ocean contaminant levels (DFO, 2008; NMFS, 2008). Shipping and boating noise disrupts foraging behaviour (Williams *et al*., 2006), pod communication (Williams *et al*., 2014) and echolocation clicks used to hunt prey (Holt, 2008). However, reducing the speed of ships, relocating major shipping routes and removing noisier ships and replacing them with newer, quieter ships would reduce the intensity of sound pollution (Williams *et al*., 2019b). Although anthropogenic sound pollution is a threat to many cetaceans, this sense can also be exploited to help reduce bycatch of these species in commercial fisheries, or to reduce pinniped predation on fish farms and fisheries. Indeed, acoustic alarms effectively reduce bycatch of harbour porpoises (*Phocoena phocoena*) without reducing target Atlantic cod (*Gadus morhua*) catches in Danish gillnet fisheries (Larsen and Eigaard, 2014). Finally, Götz and Janik (2013) have shown how acoustic deterrent devices, and specifically those that capitalize on the startle reflex system, may be effective pinniped deterrents from fish farms and fisheries, thus reducing human–wildlife conflicts in this example.

### Fish

Fish are capable of detecting a range of sound frequencies through the inner ear (Popper and Fay, 2011), as well as a range of low-frequency vibrations and mechanical disturbance (hydrodynamic stimulation) using specialized receptors in their lateral line (Bleckmann and Zelick, 2009; Higgs and Radford, 2016). This allows fish to detect distant motion and vibrations through specific mechanoreceptors. Hearing is important for many fish species for school cohesion, mate choice and spawning, finding suitable habitats (e.g. detection of ‘reef sounds’) and territory defense (Putland *et al*. 2019).

Sound pollution from boats, windfarms and hydroelectric facilities can cause a number of problems for certain fish species (Slabbekoorn *et al.*, 2010). Both marine and freshwater fishes are affected by sound from boats, ships, offshore windfarms and hydroelectric facilities (Mickle and Higgs, 2018; Popper and Hawkins, 2012, 2019). Compared to marine fishes, the effects of sound pollution on fish in the freshwater environment have been less well studied, but these species still face important threats from hydroelectric facilities and are affected by sound pollution from boats (Graham and Cooke, 2008; Mickle and Higgs, 2018; Rountree *et al*., 2020). Sound has been shown to have negative effects on the behaviours of various fish species, often causing a decrease in foraging behaviour (Codarin *et al*., 2009; Purser and Radford, 2011; McLaughlin and Kunc, 2015; Sabet *et al*., 2015). Exposure to sound pollution also causes physiological stress. For example, intermittent noise elicited a stress response in the giant kelpfish (*Heterostichus rostratus*), although the stress response was not seen when kelpfish were exposed to a constant source of sound indicating that the variability in sound pollution may be a more important factor than its presence alone (Nichols *et al*., 2015). Ambon damselfish (*Pomacentrus amboinensis*) exposed to motorboat noise also show signs of physiological stress and, as a result, have a reduced ability to evade predators (Simpson *et al*., 2016).

Sound can be strategically used to deter fish from certain areas. As previously mentioned, turbines pose a significant threat to fish who could be severely injured or killed by the fast-moving turbine blades or screws. Barriers of sound, light and bubbles can be effective at deterring fish movement through dangerous dam structures (Nestler *et al*., 1992; Ross *et al*., 1995; Noatch and Suski, 2012). Sound barriers can also be effective at controlling the spread of invasive Asian carps (*Hypophthalmichthys nobilis*, *H. molitrix*) in North America, including into the Laurentian Great Lakes (Ruebush *et al*., 2012). These invasive species are exerting generally negative effects on native communities, and thus control of their distribution could be very beneficial to ecosystem functioning. Sound barriers also blocked movement of native fish species present in the area, which nay negatively impact population processes within resident communities. Sound is being investigated as a deterrent for invasive sea lamprey in the Great Lakes basin and to date, low-frequency sounds have induced the strongest behavioral responses (Mickle*et al*., 2019).

### Invertebrates

For insects, acoustic stimuli are very important for intraspecific communication, predator avoidance and prey detection, and as a result hearing has evolved multiple times in parallel across seven insect orders (Hedwig, 2014). Insects have developed tympanal ears, characterized by a membrane (tympanum) that vibrates in response to sound (Hoy and Robert, 1996), and these ears can be found on various body parts in different insect species (Hoy and Robert, 1996; Göpfert and Hennig, 2016). Other invertebrates, such as arachnids and crustaceans, do not have tympanal ears, although some species may still be able to detect certain sound frequencies but relatively little is known (Barth, 2000; Stumpner and von Helverson, 2001; Edmonds *et al*., 2016). However, for many arthropods, substrate vibrations are an important stimulus (Hill, 2008). For example, spiders exploit substrate vibrations for multiple purposes including mating behaviour whereby spiders send out vibrations through leaf litter (Uetz and Roberts, 2002), and detecting vibrations in their webs (Barth *et al*., 1988; Landolfa and Barth, 1996). Although we found no case studies demonstrating the successful application of invertebrate auditory ecology for conservation purposes, we do highlight important research on invertebrate hearing and problems resulting from anthropogenic activity and resulting sound pollution.

Invertebrates, both aquatic and terrestrial, are affected in numerous ways by sound pollution. Noisy compressor stations at oil and natural gas facilities have been shown to have negative consequences on invertebrate populations. One study found the abundance of a number of different arthropod species to be negatively associated with noisy compressor sites, and this might have significant knock-on impacts for the surrounding ecosystem (Bunkley *et al*., 2017). In the marine environment, zooplankton are integral to the productivity of the ocean as the primary food source for a vast array of marine species, including many species of fish and cetaceans. One study found that seismic surveys, an acoustic imaging technique used to search for petroleum in the ocean, can cause significant mortality for zooplankton (McCauley *et al*., 2017). In particular, the abundance of zooplankton decreased by 64% following an acoustic impulse signal, affecting zooplankton up to 1.2 km away from the signal source. Based on these findings, seismic surveys might be having significant negative impacts on ocean ecology that is not widely acknowledged.

As well as effects on abundance, sound pollution can affect the behaviour of invertebrates. For example, bow-winged grasshoppers (*Chorthippus biguttulus*) found near loud roadways (Lampe *et al*., 2012) and the cicada (*Cryptotympana takasagona*) found in louder environments (Shieh *et al*., 2012) both emit higher frequency calls than their conspecifics in quieter environments. This is an adaptation to prevent masking of their auditory calls to potential mates, highlighting that sound pollution can have negative implications for mating and reproductive success. Indeed, another study found that traffic noise resulted in failure of female field crickets (*Gryllus bimaculatus*) to orient to male auditory calls (Schmidt *et al*., 2014). However, the failure of females to orient may not have been the result of male auditory signal masking, but instead because females were distracted by traffic noise.

Distraction, in addition to masking and stress, is also a potential consequence of acoustic noise (Chan and Blumstein, 2011). Studies on terrestrial Caribbean hermit crabs (*Coenobita clypeatus*: Chan *et al*., 2010) led to the development of the distracted prey hypothesis (Chan and Blumstein, 2011). The hypothesis notes that any stimulus that can be detected has the potential to re-direct the limited attention that a species has, and this can have negative consequences for risk assessment. For hermit crabs the sounds of boat motor noise modified risk assessment by permitting humans to get closer to individual crabs before they retreated into their shells.

## Chemoreception

Both biotic and abiotic components within an environment release molecules and chemical compounds that can provide information to individuals. Animals are often able to detect chemical stimuli through olfaction, gustation and chemesthesis. Olfaction is the ability to detect (chemical) odours without physical contact with the source (Eisthen, 2002) and is often a vital component of reproductive and social behaviours, individual or group recognition, as well as predator–prey interactions. Chemesthesis is the detection of chemical stimuli via receptors and neurons found in the integument of animals (Slack, 2016). Gustation (or taste) also involves the detection of chemicals or molecules but uses different families of chemoreceptors and different signalling pathways to the brain (Wyatt, 2014). Through gustation animals perceive chemical stimuli as tastes or flavours, whereas through olfaction they are perceived as smells. Chemical stimuli can be broadly categorized as environmental odors (chemical stimuli from abiotic sources such as water, fire, soil types or habitats) and semiochemicals (produced by other animals for the purpose of inter- or intraspecific interactions). Semiochemicals can be further categorized as pheromones, signature mixes and allelochemicals. Pheromones are involved in intraspecific communication and elicit an innate response for a specific purpose, such as mating, alarm cues or mother–offspring interactions (Wyatt, 2010). Signature mixes, on the other hand, are variable chemical mixtures that are learned, and typically allow an animal to identify an individual or social group (Wyatt, 2010). Finally, allelochemicals are important for interspecific interactions and may function in various ways that benefit the emitter, the receiver or both (Wyatt, 2014).

In this section, we will discuss how chemosensory ecology, in particular olfaction and gustation, can be exploited in conservation and wildlife management. Research has led to the development of highly effective animal control techniques through creating species-specific olfactory traps. Examples include controlling pest, invasive or overabundant species, and mitigating human–wildlife conflicts. The majority of the literature on pest control through exploiting olfactory systems focuses on insects as various species are common pests in agriculture and vectors for diseases, and thus there is a strong need to control their populations in some instances (Witzgall *et al*., 2010). However, invasive species of other major taxa have also been successfully managed through exploiting their olfactory sense. Olfactory traps can also be exploited by wildlife managers to help control overabundant, or invasive populations that might have negative effects on an ecosystem, or overabundant predator populations, which cause further threat to endangered prey populations (Baker, 2009; Cruz *et al*., 2009; Johnson *et al*., 2009). Unfortunately, human activity is also causing unwanted, inadvertent olfactory traps in environments that have negative effects on certain species. There are case studies of this in marine environments causing harmful effects on fish and birds (Savoca *et al*., 2016; Williams *et al*., 2019a).

### Birds

It was once thought that birds were anosmic or have very limited olfactory capabilities. However, furthering research into avian olfaction has revealed a great importance for this sense across many bird species (Balthazart and Taziaux, 2009; Prada and Furton, 2018). Birds rely on olfaction for a number of purposes, including searching for food (Wenzel, 1968; Graves, 1992; Nevitt, 2000; Nevitt, 2008), homing behaviour and navigation (Wallraff, 2004; Gagliardo, 2013) and for nest localization (Bonadonna *et al*., 2003; Bonadonna and Nevitt, 2004). In particular, certain species of seabirds rely heavily on olfaction (Nevitt, 2008). Plastic waste in the ocean is often ingested by seabirds, but the reasons why have been unclear. However, a recent sensory physiology study has begun to explain this common ecological trap. Savoca *et al.* (2016) showed that microplastics that have been in the marine environment for extended periods of time produce dimethyl sulfide (DMS). DMS is also a common odorant that is released by the prey of many seabirds, thus causing seabirds to ingest plastic mistaking it as a viable food source. Plastic is also ingested by a number of other marine animals, including species of sea turtles and whales, and further research is necessary to determine whether DMS released from these plastics also acts as an olfactory trap for these species (Savoca *et al*., 2016).

Behaviour of certain species can be manipulated through taste conditioning. Conditioned taste aversion (CTA) is one such method used to manipulate the behaviour of an individual. Through this method, individuals are taught to associate certain food items with a negative taste experience. True CTA has been successfully used to reduce predation of endangered bird eggs. In one study that aimed to reduce red fox (*Vulpes vulpes*) predation on threatened hooded plover (*Thinornis rubricollis*) eggs, model eggs that mimicked those of the hooded plover were produced and treated with a CTA-inducing chemical (Maguire *et al*., 2009). Control eggs were also produced without the CTA chemical treatment and placed in the experimental setting. This study found CTA to be successful in reducing predation on both treated and control eggs, showing promise for CTA as an effective way to deter predators and protect endangered species. CTA has also been successfully implemented in this way to reduce predation by grey wolves (*Canis lupus*; Gustavson, 1982), coyotes (*C. latrans*; Ellins and Catalano, 1980) and brushtail possum (*Trichosurus vulpecula*; Clapperton *et al*., 1996), and to reduce crop damage by African elephants (*Loxodonta* spp.; Osborn, 2002).

### Mammals

For mammals, the main olfactory organ is the nose, within which there are many olfactory subsystems. In particular, the main olfactory epithelium and vomeronasal organ are the most widely studied and contain different classes of receptors (Trotier, 2011; Wackermannová *et al*., 2016). Mammals rely heavily on olfaction during mating, locating food, avoiding predators and for individual recognition and social behaviour. Pheromones are widely used for communication in mammalian species, for example in mating, territorial defense, alarm signals and mother–offspring interactions (Brennan, 2010). During mating, some mammalian females release pheromones to attract male conspecifics, and this knowledge can be exploited for conservation purposes. Due to the strong, innate response mating pheromones often elicit, the use of pheromones can be highly effective at controlling invasive, destructive mammalian populations, as we will discuss here. Mammals are also capable of detecting chemical stimuli through gustation (or ‘taste’). There are five taste modalities that chemical stimuli can be categorized through gustation: sweet, bitter, sour, salty and umami (Yarmolinsky *et al*., 2009). Bitter and sour tastes are typically ‘bad’ tastes and alert the animal to harmful foods, for example toxins, noxious plants and spoiled food. Although we will not discuss gustation in the context of mammalian conservation in this section, mammalian gustation can be exploited for the conservation of other taxa (e.g. birds) as was previously discussed.

Common goat (*Capra hircus*) females release sexpheromones that attract males for reproduction purposes. These goats were introduced by humans to the Galapagos archipelago where they quickly established a fast-growing population that had destructive effects on native biota and eventually demanded eradication of the population (Robertson *et al*., 2017). Initially, large numbers of the goats were removed by ground and aerial hunting. However, when the goat population reached low density, they became increasingly difficult to hunt. The remaining goats were then eradicated through a very effective sensory trap. Female goats were captured, sterilized and put in a chemically induced estrus, which caused them to produce pheromones that were detected by males as an attractant for mating. Males mated with these sterilized ‘Judas’ goats, resulting in no offspring. This technique eventually resulted in complete eradication of the invasive goat population on certain Galapagos Islands (Cruz *et al*., 2009), which greatly helped restoration of native flora. In this case study, male goat olfaction was exploited by creating a sensory trap to eradicate an invasive population. Other eradication techniques had been attempted before implementing this sensory trap, but they were not successful at complete eradication of the goat population, thus highlighting the advantages of the sensory ecology approach to invasive species management. In Australia, feral goats (*Capra hircus*) were controlled through predator olfactory deterrents. Cox *et al.* (2012) demonstrated that feral goats avoided areas with dingo (*Canis lupus dingo*), lion (*Panthera leo*) and tiger (*P. tigris*) odors, which could have implications to shift goat grazing areas to other areas where they may not be causing as much damage or competition with endangered native species. Olfactory predator deterrents have also been shown to be successful with a number of other species including certain marsupials (Parsons and Blumstein, 2010) and vervet monkeys (*Cercopithecus aethiops*; Willems and Hill, 2009).

Olfaction can also be exploited to support at-risk species. For example, urine scent has been shown to influence mate choice in female pygmy loris (*Nycticebus pygmaeus*), a threatened primate species (Fisher *et al*., 2003). This knowledge can be used to promote successful mate pairings that maximize genetic compatibility and diversity to aid in supporting healthy populations. In another example, improved conservation breeding in captivity or translocation programs could be achieved by familiarizing females with male scents to reduce aggression and increase breeding success, as demonstrated with harvest mice (*Micromys minutus*: Roberts and Gosling, 2004).

### Fish

Fish detect chemical stimuli in the aquatic environment and rely on these cues for reproduction, feeding, alarm (Smith, 1992) and, in some species, vast migrations (Yamamoto *et al*., 2010; Bett *et al*., 2016; Sorensen and Johnson, 2016). Fish are capable of detecting chemical stimuli through olfaction, gustation and solitary chemosensory cells (SCCs; Hansen and Reutter, 2004). SCCs occur on the skin, gills and oropharynx of fish and have been suggested to function to locate food, predators or conspecifics (Sbarbati and Osculati, 2003; Hansen and Reutter, 2004). For fish, olfaction is a ‘distance’ sense, allowing fish to detect chemical stimuli from conspecifics, food, predators and even habitats at sometimes great distances. For example, olfaction is important in long distance spawning migrations, as demonstrated by salmonid fishes as reproductive adults navigate from marine or lacustrine foraging environments to their natal tributary streams following very dilute stream-specific chemical odours over great distances (Yamamoto *et al*., 2010; Bett *et al*., 2016). It is important that salmonid populations return to their specific natal streams because their eggs are genetically programmed with specific incubation times and growth rates adapted to the specific environmental conditions of that stream (Dittman and Quinn, 1996). Straying of salmonids to spawn in non-natal streams will often result in death of the offspring due to mismatches between environmental conditions and localized adaptations (Bett *et al*., 2017). Elevated carbon dioxide concentrations in seawater can disrupt the olfactory senses that salmon rely so heavily upon to navigate to natal streams (Williams *et al*., 2019a). Knowledge of the sensory implications of increasing ocean acidification, an unfortunate result of global climate change, offers more insight into yet another threat faced by this species.

Sex pheromones in species of invasive fish have been successfully exploited to control populations of some species. Sea lamprey (*Petromyzon marinus*) are invasive species to the Laurentian Great Lakes and are destructive parasites of valued sport fishes. Researchers found that male sea lamprey release a mixture of sex pheromones and identified several components (Buchinger *et al*., 2015). The major component has been synthesized and attracts ovulated females into baited traps from over hundreds of meters (Johnson *et al*., 2009). These studies of pheromone- or sensory-based pest control could provide a highly effective, species-specific way of controlling invasive or harmful species. This example of control of sea lamprey with sex pheromones represents the first of its type demonstrated in a vertebrate species. Further research in this field could lead to more sensory-based pest control techniques in a range of other species and taxa.

### Invertebrates

Most invertebrates rely heavily on olfaction and as a result have evolved very sensitive olfactory systems (Hildebrand and Shepherd, 1997). This is a very broad, diverse topic across all invertebrate species; however, we will only focus specifically on insect olfaction as this field of research has shown most relevance to conservation and management. Many invertebrate species use pheromones for communication and mating, and these pheromones will often elicit a strong innate response. Farmers and conservationists can therefore exploit this innate response to lure insects to sensory pheromone traps, and there are now numerous examples of how insect species can be controlled and monitored in this way (El-Sayed *et al*., 2006; Baker, 2009). This so-called ‘mass trapping’ uses specific chemical lures, usually sex aggregation pheromones or food odors, to attract certain insects to lethal traps (El-Sayed *et al*., 2006). Many scientific studies describe the use of mass trapping for control of pest insect species, for example in agricultural practices; however, these pheromone traps are also beneficial in conservation science for monitoring endangered species. Numerous examples illustrate the use of pheromones to attract endangered insects (e.g. Millar*et al*., 2010; Ray *et al*., 2014; Konig *et al*., 2016). Pheromone-based trapping is highly beneficial for monitoring populations and sampling of threatened species because it is highly species specific and effective at even low population densities (Larsson, 2016). Chemical ecology has also been exploited for the control of destructive, invasive insect species that devastate native flora and fauna (Smith, 1998; El-Sayed*et al*., 2006; Larsson, 2016). The gypsy moth (*Lymantria dispar*) is an invasive species in the United States that feeds on many woody plants and has devastating effects on forests (Davidson *et al*., 1999) and there is therefore a need to control numbers of this species. Pheromone trapping of insects can be a highly effective conservation tool, allowing us to both monitor endangered species, and control invasive species, to conserve native flora and fauna (Tobin and Blackburn, 2007).

## Other Sensory Modalities

### Electroreception

Electroreception is defined as the detection of electric information within the environment (Stevens, 2013). Water, unlike air, is a good conductor of electricity and thus species that are capable of electroreception typically live in aquatic or moist environments. This sensory modality is particularly widespread in fishes and amphibians (Crampton, 2019) but is also important in other taxa. Studies have demonstrated the importance of electroreception in mammalian species such as the platypus (*Ornithorhynchus anatinu*; Scheich *et al*., 1986), star-nosed mole (*Condylura cristata*; Gould *et al*., 1993) and Guiana dolphin (*Sotalia guianensis*; Czech-Damal *et al*., 2012), and also aquatic invertebrates such as the crayfish (*Cherax destructor*; Patullo and Macmillan, 2007; Patullo and Macmillan, 2010) and fish species including sea lamprey (Chung-Davidson *et al*., 2004; Johnson *et al*., 2016).

Electroreception, like audition, can be passive or active. Passive electroreception is the ability to detect weak direct current fields or low-frequency sinusoidal fields (Crampton, 2019). These electric stimuli are detected by highly specialized cells (electroreceptors) located in pores of the animal exposed to water (Peters *et al*., 2007). Passive electroreception is important for detection of prey in several aquatic species, such as the spotted dogfish (*Scyliorhinus canicular*) that can detect its prey, the flatfish (*Pleuronectes platessa*), using only electrical stimuli (Kalmijn, 1971). Active electroreception is less common than passive electroreception. It can be defined as the ability of certain animals, in particular teleost fishes, to produce electric fields and measure distortion of these fields by surrounding objects in the environment (Alvez-Gomes, 2001). These species are defined as electrogenic, and they create electric fields through specialized muscle fibres that consist of electrocytes (Stoddard and Markham, 2008). These electrocytes are capable of polarizing the skin of the fish, which creates a surrounding electric field. Any objects close to the fish will then cause distortions in this electric field, which, in turn, will be detected by electroreceptors on the fish allowing it to localize surrounding objects and individuals (Von der Emde, 1999). Active electroception has been shown to be important for communication (Dunlap *et al*., 2010), mating (Curtis and Stoddard, 2003), recognition (Nagel *et al*., 2018) and hunting (Hanika and Kramer, 2000).

Understanding electroreception may have applied benefits as seen in successful case studies that effectively reduced shark bycatch by exploiting their electrorepulsive behaviour (Brill *et al*., 2009; Hutchinson *et al*., 2012). A major threat to many aquatic species is bycatch. Commercial fisheries often deploy large nets or trawl fishing hooks to catch large numbers of target fish species. However, non-target species can also be caught by these commercial fishing practices and bycatch of sharks in this way has contributed to mortality and declining numbers of certain species (Ferretti *et al*., 2008; Molina and Cooke, 2012; Gallagher *et al*., 2014). Sharks are capable of detecting electric fields, whereas target fish species often cannot, leading to the use of electropositive metals on fishing hooks (O'Connell *et al*., 2014) and reduced shark catch rates (Hutchinson *et al*., 2012). This result appeared to be species specific, however, as the electropositive hooks reduced bycatch of hammerhead sharks (*Sphyrna lewini*) but not sandbar sharks (*Carcharhinus plumbeus*: Hutchinson*et al*., 2012).

### Magnetoreception

Magnetoreception is the ability to sense the earth’s magnetic field, but is not as well understood as other sensory modalities (Wiltschko and Wiltschko, 2005; Lohmann *et al*., 2007; Gould, 2010; Wiltschko and Wiltschko, 2012; Mouritsen, 2018). Magnetoreception has been shown across most taxa and is exploited for orientation and navigation. Because magnetic fields pass through animal tissues, identifying receptors and understanding the physiological basis of detection have been difficult (Walker *et al*., 1997; Johnsen and Lohmann, 2005). However, there are now several proposed mechanisms of magnetic field detection in various animals including highly specialized electroreceptors, magnetic-particle-based magnetoreception and radical-pair-based magnetoreception (Mouritsen, 2018).

Magnetoreception is important for migratory birds (Wiltschko and Wiltschko, 2005) and insects such as the monarch butterfly (*Danaus plexippus*; Guerra *et al*., 2014). Magnetoreception is also important for shorter-distance movements in birds, for example in homing behaviour of pigeons (*Columba livia f. domestica*: Wiltschko *et al.* 2010), and in home range navigation of domestic chickens (*Gallus gallus*: Wiltschko *et al*., 2007). Animals such as rodents and migratory salmonids do not use an inclination-based compass, but instead use a ‘polarity compass’, whereby they are able to detect the polarity of the field lines allowing them to perceive north and south (Wiltschko and Wiltschko, 2005). This compass is important in long-distance migrations as smolts heading to the ocean (Quinn and Brannon, 1982), and again as adults returning to natal streams (Putman *et al*., 2013; Lohmann and Lohmann, 2019). A third and final mechanism of using magnetic fields for navigation is known as ‘magnetic maps’, where an individual can determine its position relative to a target location for migration (Lohmann *et al*., 2007). This type of navigation by magnetoreception is used by spiny lobsters (*Panulirus argus*; Boles and Lohmann, 2003), and various species of sea turtles (Lohmann *et al*., 2007).

Magnetoreception has been clearly demonstrated to be a vital sense for orientation and navigation in a growing number of species. However, compared to other sensory modalities, it has been relatively under-studied and there are still many questions concerning the sensory ecology and physiology of magnetoreception. However, in recent years, the impacts of human electromagnetic noise on animal magnetic compasses have been investigated, and researchers have documented interfering effects of such noise on bird magnetic orientation. Electromagnetic noise from human activity has been shown to disrupt magnetic compass orientation in European robins (*Erithacus rubecula*) in both natural and labortary settings (Engels *et al*., 2014; Schwarze *et al*., 2016). These findings may suggest that growing anthropogenic development may be causing widespread negative effects on bird migration and may represent a more serious conservation issue. Other studies have since raised concern that electromagnetic noise may be having similar disruptive effects on the magnetic compass of other animals, such as the monarch butterfly (Guerra *et al*., 2014; Reppert *et al*., 2016); however, there is no evidence to support this yet. Anthropogenic activity may also be affecting animal magnetoreception in ways that we are not yet aware of, and furthering research into this sensory modality may reveal potential sensory traps, or ways conservation practioners can exploit magnetoreception for applied purposes.

### Multimodal stimuli

Animals often receive and process multimodal stimuli (Partan and Marler, 1999; Munoz and Blumstein, 2012; Munoz and Blumstein, 2020). Multimodal cues and signals target multiple sensory systems in the receiver. The receiver must then integrate this multisensory information to make decisions and modify behaviour appropriately (Munoz and Blumstein, 2012). Multimodal signalling is thought to have evolved to either increase the information content in a signal (i.e. non-redundant signalling whereby different sensory components of a signal provide different information to the receiver) or to increase the robustness of the signal reaching the receiver (redundant signalling whereby each component of the signal provides the receiver with the same information; Hebets and Papaj, 2005; Partan and Marler, 2005). For example, non-redundant multimodal begging signals from reed warbler (*Acrocephalus scirpaceus*) offspring provide their parents with increased information (Kilner *et al*., 1999). The area of the visual gape displayed by the brood provides information on the age and size of the brood, whereas the vocal calls provide information on the hunger levels of the brood, and both signals allow parents to adjust their feeding rates accordingly. We see an example of redundant multimodal signalling during wolf spider (*Schizocosa stridulans*) courtship behaviour that includes both visual and seismic signals (Hebets, 2008). Visual signals, although not necessary for mating success, are used as they can travel farther than seismic signals and thus increase the probability of being detected. However, when mates come closer, seismic signals become a dominant and necessary signal for mating success. In this example the multimodal components of the signal are redundant, both providing the same information to the receiver but increasing the probability the signal will be received (see Partan and Marler, 1999; Partan and Marler, 2005; Munoz and Blumstein, 2012; Higham and Hebets, 2013; Stevens, 2013).

Studying multisensory integration is complex and requires a number of considerations. Animals may not use all available stimuli, sometimes only responding to one sensory cue or signal. The sensory information that an animal uses may also change depending on various factors such as reproductive state (Kasurak *et al*., 2012), environmental conditions (Munoz and Blumstein, 2020) and seasonality (Gall *et al*., 2013). For example, one study found that female round gobies (*Neogobius melanostomus*) only integrated vibrational and olfactory sexual stimuli from males when they are reproductive, which aids in finding the specific location of reproductive males and nest sites (Kasurak *et al*., 2012). Interspecific multisensory integration adds another level of complexity where we must now consider the different sensory systems, sensory thresholds and cognitive abilities of various species (Munoz and Blumstein, 2020). Interspecific multimodal signaling is important in aposematism (Rowe and Guilford, 1999), for example, the warning signals of seven-spot ladybirds (*Coccinella septempunctata*) are multimodal signals comprising visual and chemical warning signals (Marples *et al*., 1994). In another example, plant–pollinator interactions benefit from interspecific multimodal signaling, as we see multimodal signals enhance decision making in common eastern bumble bees (*Bombus impatiens*), allowing individuals to learn and detect more rewarding flowers faster (Kulahci *et al*., 2008). Even intraspecific multisensory integration is relevant in mate choice contexts as shown by female brown-headed cowbirds: females with better auditory temporal resolution prefer shorter and high frequency male songs, whereas females with better temporal visual resolution prefer less intense male visual displays (Ronald *et al*., 2018). Understanding and predicting animal integration of multisensory cues and signals often require complex models and frameworks. Not only do we need to predict what sensory cues and signals an animal will respond to at a particular time, but also how that animal will respond in its given condition and environment. There are now several models and frameworks that can be applied to multimodal signalling in animals and have helped further our understanding of this complex signalling (see Munoz and Blumstein, 2012; Wilkins *et al*., 2015; Hebets *et al*., 2016; Munoz and Blumstein, 2020).

There are a few studies that have focused on the impacts of anthropogenic activity using a multisensory/multimodal perspective. Rabin *et al.* (2006) studied the effects of auditory noise from wind turbines on California ground squirrels (*Spermophilus beecheyi*), finding that squirrels were less responsive to auditory predator signals, and instead increased their alertness to obtain more visual cues. Partan *et al.* (2010) found a similar multimodal shift from reliance on audio to visual cues in the eastern grey squirrel (*Sciurus carolinensis*). Assessing how human development, and planned development, affects multisensory perception in animals will allow a more comprehensive measurement of the impacts of such development on affected animals (Munoz and Blumstein, 2012; Partan, 2017). In a recent study, researchers found multisensory stimuli may have beneficial implications for controlling sea lamprey movement. Johnson *et al*. (2019) studied the impacts of visual and olfactory stimuli applied together on juvenile sea lamprey movement. Results showed that light attractant stimuli, and conspecific odorant repellent stimuli, can be used together in a ‘push and pull’ configuration to better control movement of these fish. Such findings of exploiting multisensory attractants and repellents together could be useful to more effectively reduce turbine entrainment of native species, or instead to increase trapping of invasive species. Understanding multisensory perception may also be beneficial to improving translocation and reintroduction programs designed to help recover species by returning them to suitable available habitat. Translocation, reintroduction and captive release of species are often unsuccessful, and failure of animals to detect and recognize various stimuli in their new environment is frequently attributed to these low success rates (Stamps and Swaisgood, 2007). For example, introduced species may fail to recognize the chemical scent or auditory calls of suitable prey or fail to identify and evade predators, thus resulting in unsuccessful introduction. Understanding the multisensory perceptions of an animal in its habitat may help increase the success rates of introducing these animals to novel habitats (Munoz and Blumstein, 2012). Finally, many unimodal repellents fail (Lecker *et al*., 2015). Often this is because animals habituate to unreinforced unimodal threat stimuli. There have been suggestions that by designing multisensory repellents, habituation can be delayed (Lecker *et al*., 2015).

## Benefits, challenges and future directions for applied sensory ecology

### Benefits

Sensory-based approaches can be effective in conservation interventions, and throughout this paper we see recurrent applications of sensory ecology for certain conservation challenges. Through an extensive review of the literature, we have highlighted many successful applications of sensory ecology to conservation and wildlife management (Table 1), as well as a large amount of research furthering the field of sensory ecology with potential to benefit conservation science (Table 2). In particular, sensory approaches can be beneficial, and successful, for ameliorating sensory traps, reducing harm to wildlife (i.e. from bycatch or airstrikes), and wildlife relocation. Through emphasizing these success stories, and discussing the benefits, challenges and future potential of sensory ecology, we hope to encourage wildlife managers and conservationists to consider sensory-based approaches to conservation issues. We also highlight gaps in the literature to encourage future research and development in the fields of applied sensory ecology and sensory physiology.

Many conservation issues are a consequence of human activity, such as rapid environmental change, habitat loss or climate change (Sutherland *et al*., 2019; Sutherland *et al*., 2020). As these issues continue, it is important to predict how species will respond to such environmental changes, which species will be most susceptible, and ultimately how to protect and restore those at risk. Sensory approaches are needed to more accurately predict how, why and when human activities may threaten wildlife populations. Such approaches avoid biases of human perception and can predict which types of environmental changes are actually perceived as stressful by wildlife, and how various species might respond to these changes. Further, research identifying potential sensory traps can help guide development and construction of human structures (e.g. building facades, wind turbines) and equipment (e.g. fishing gear) towards more wildlife-friendly options. For example, new beach developments in important turtle habitat should use specific lighting to prevent misguiding hatchlings (Witherington *et al*., 2014), while future solar farm constructions should consider new designs that are less attractive to aquatic insects (Horváth *et al*., 2010). As the human population continues to grow, and development expands across the globe, identifying and developing solutions to prevent sensory traps will be highly beneficial in protecting species into the future.

### Challenges

Many challenges remain for integrating sensory ecology with wildlife conservation and management. Environmental heterogeneity, both natural and anthropogenic, makes it difficult to understand or predict with high degrees of accuracy what an animal may perceive in nature (Dangles *et al.*, 2009). For example, what an animal can see can be influenced by air or water clarity when detectability and acuity of visual cues are reduced under conditions of fog or turbidity (air and water, respectively). Similarly, wind and water velocity (current) limits the ability to detect and identify cues, as well as locate cue sources. Individual variability in sensory sensitivity adds another layer of complexity (Dangles *et al.*, 2009): just as some humans have better eyesight, hearing or smell than others, the same is true within and between other species (e.g. Ronald *et al.*, 2017). Sensory physiology studies can be logistically challenging, and some questions are best addressed under laboratory conditions where background noise can be minimized (Dangles *et al.*, 2009). Researchers with the understanding and ability to conduct sensory physiological assays in laboratory environments may not have the expertise to do so in ecological field environments and vice versa, creating a communication barrier between lab and field researchers. However, sensory physiology interfaces with many disciplines and can connect physiologists, ecologists and practitioners to maximize research efficiency and benefit research and management efforts (Caro and Sherman, 2013; Lennox and Cooke, 2014).

### Future Directions

The growing number of success stories emerging from the integration of sensory ecology with conservation and wildlife management are promising signs that highlight the benefits of studying the sensory perceptions of species of interest. Increasing exposure and emphasis on these success stories is hoped to encourage conservationists and wildlife managers to consider sensory approaches to solve conservation issues in the future, and perhaps even encourage funding opportunities for such interdisciplinary research. Greater exposure of sensory-based conservation science can be achieved through review articles, conference presentations (Madliger et al., 2017) and integration of this cross-disciplinary field at graduate and undergraduate levels.

Throughout this paper we highlighted a large amount of research furthering our understanding of species sensory ecology with potential to benefit conservation sciences (Table 2). Furthering our understanding of what organisms perceive, and which types of sensory stimuli are causing harmful or negative effects on organisms (i.e. artificial light at night, noise pollution, etc.), will be necessary for managers to know where future regulation or management changes are needed to protect wildlife. For example, there are a number of studies documenting the negative effects that noise pollution can have for many species across taxa (Table 2). There is great potential for managers and decision-makers to make regulation changes that benefit wildlife based on these findings, for example rerouting of major shipping routes to avoid important whale habitat (Williams *et al*., 2019b), or development of quieter compressor stations to reduce stress to wildlife (Habib *et al*., 2007; Bunkley *et al*., 2015; Kleist *et al*., 2018). Table 2 provides recommendations for future conservation and management decisions, based on a sensory understanding, that could benefit wildlife. We also recommend that furthering research into sensory ecology of species of interest will help to highlight other conservation problems that can begin to be addressed.

Despite a relatively small amount of research in the field of applied sensory ecology, we do observe certain taxonomic biases (Fig. 1). Specifically, we note research biases towards avian and mammalian species, a common observation in the field of conservation (Donaldson *et al*., 2016; Troudet *et al*., 2017). Amphibian sensory ecology is largely understudied when compared with other vertebrate groups and we identified no research in this field currently being applied to conservation. Reptiles are also relatively understudied in sensory ecology, and we identified endangered green sea turtles (*Chelonia mydas*) as the only species for which sensory ecology has been studied with applications for conservation. Donaldson *et al*. (2016) also reported green sea turtles as an outlier in reptile biodiversity conservation papers, again highlighting bias towards this reptile species. Despite invertebrates also being largely understudied compared with vertebrate groups in conservation fields (Donaldson *et al*., 2016; Troudet *et al*., 2017), we found a number of invertebrate sensory ecology studies with potential benefits to conservation (Table 2), and one demonstrated application of aquatic insect visual ecology ameliorating a sensory trap (Horváth *et al*., 2010). However, we recognize that compared with all vertebrate taxonomic groups discussed in this paper, invertebrates were again relatively understudied. Furthering sensory research for understudied taxa could provide additional benefits to the conservation and protection of these species and reduce taxonomic biases in this field.

To improve communication and collaboration across sensory ecology, physiology and conservation science fields there is a need for increased research efforts demonstrating sensory-based conservation strategies, and a clear understanding of conservation needs (see Greggor *et al*., 2016 for and in-depth discussion into resolving this interdisciplinary communication issue). Relating to sensory ecology, further research into multimodal stimuli (e.g. Partan *et al*., 2010; Johnson *et al*., 2019), multi-species approaches (e.g. Spoelstra *et al*., 2015) and linking sensory physiology to behaviour (e.g. Sillman *et al*., 2007; Fernandez-Juricic *et al*., 2013; Elvidge *et al*., 2019) would benefit conservation.

## Conclusions

Every organism lives in its own sensory world, each perceiving the environment through different sensory organs. Knowledge of how and what different organisms perceive has helped us understand seemingly counter-intuitive, maladaptive behaviours including birds colliding with moving vehicles (DeVault *et al*., 2015), aquatic insects laying eggs on solar panels (Horváth *et al*., 2010) and marine animals ingesting plastics (Savoca *et al*., 2016). Furthering our understanding of these sensory problems can lead to solutions on how to resolve and prevent them in the future. As we have demonstrated through numerous case studies, sensory ecology has proven to be a valuable and effective tool in wildlife conservation and management, and we have generated suggestions for where and when mechanistic studies of perceptual mechanisms may provide informative insights. Promotion and exposure of the benefits sensory ecology can provide for conservation sciences is needed, and conservationists and wildlife managers are encouraged to consider sensory-based approaches to conservation issues.
